# Laser-assisted rapid evaporative ionisation mass spectrometry (LA-REIMS) as a metabolomics platform in cervical cancer screening

**DOI:** 10.1016/j.ebiom.2020.103017

**Published:** 2020-09-25

**Authors:** Maria Paraskevaidi, Simon J.S. Cameron, Eilbhe Whelan, Sarah Bowden, Menelaos Tzafetas, Anita Mitra, Anita Semertzidou, Antonis Athanasiou, Phillip R. Bennett, David A. MacIntyre, Zoltan Takats, Maria Kyrgiou

**Affiliations:** a Department of Metabolism, Digestion and Reproduction & Department of Surgery and Cancer, Faculty of Medicine, Imperial College London, 665 Sir Alexander Fleming Building, South Kensington Campus, London W12 0NN, United Kingdom; b Department of Pharmacy and Biomedical Sciences, University of Central Lancashire, Preston PR1 2HE, United Kingdom; c Institute for Global Food Security, School of Biological Sciences, Queen's University, Belfast, Northern Ireland BT5 9DL, United Kingdom; d Imperial College Healthcare NHS Trust, London, United Kingdom; e March of Dimes European Preterm Birth Research Centre, Imperial College London, London W12 0NN, United Kingdom

**Keywords:** Cervical cancer, CIN, Cervical cancer screening, Liquid-based cytology, HPV tests, Metabolomics, Laser-assisted reims (LA-REIMS)

## Abstract

**Background:**

The introduction of high-risk human papillomavirus (hrHPV) testing as part of primary cervical screening is anticipated to improve sensitivity, but also the number of women who will screen positive. Reflex cytology is the preferred triage test in most settings but has limitations including moderate diagnostic accuracy, lack of automation, inter-observer variability and the need for clinician-collected sample. Novel, objective and cost-effective approaches are needed.

**Methods:**

In this study, we assessed the potential use of an automated metabolomic robotic platform, employing the principle of laser-assisted Rapid Evaporative Ionisation Mass Spectrometry (LA-REIMS) in cervical cancer screening.

**Findings:**

In a population of 130 women, LA-REIMS achieved 94% sensitivity and 83% specificity (AUC: 91.6%) in distinguishing women testing positive (*n* = 65) or negative (*n* = 65) for hrHPV. We performed further analysis according to disease severity with LA-REIMS achieving sensitivity and specificity of 91% and 73% respectively (AUC: 86.7%) in discriminating normal from high-grade pre-invasive disease.

**Interpretation:**

This automated high-throughput technology holds promise as a low-cost and rapid test for cervical cancer screening and triage. The use of platforms like LA-REIMS has the potential to further improve the accuracy and efficiency of the current national screening programme.

**Funding:**

Work was funded by the MRC Imperial Confidence in Concept Scheme, Imperial College Healthcare Charity, British Society for Colposcopy and Cervical Pathology, National Research Development and Innovation Office of Hungary, Waters corporation and NIHR BRC.

**Research in context**

**Evidence before this study**

Human papillomavirus (HPV)-based screening has improved sensitivity compared to cytology and has replaced cytology in primary cervical screening. HPV-based screening is expected to increase the number of women that will test positive at screening due to low specificity. Currently, reflex cytology is the single validated triage test in the UK for referral to colposcopy, although this has demonstrated only moderate sensitivity and specificity. Furthermore, triage cytology lacks automation, is lengthy, prone to human error and is of low sensitivity. Given the high prevalence of passenger HPV infections with no carcinogenic potential and the mediocre performance of most existing triage tests, novel technologies that offer rapid, cost-afforded and simultaneous HPV testing and automated triaging of women at high risk of high-grade precancer are highly sought after.

**Added value of this study**: In this proof-of concept study, we have tested the use of an innovative high-throughput ambient platform, Laser-assisted Rapid Evaporative Ionisation Mass Spectrometry (LA-REIMS) in the cell pellets from liquid-based cytology samples. The results suggest that LA-REIMS can rapidly discriminate hrHPV positive from negative samples with good accuracy (94% sensitivity, 83% specificity) when compared to currently validated hrHPV tests. Furthermore, it can discriminate normal from high-grade pre-invasive precancerous cells with 91% sensitivity.

**Implications of all the available evidence**: If larger cohort and biobank studies prove high performance, this technology could offer a single, cost-effective, automated and rapid test for cervical cancer screening, obviating the need for separate screening and triage tests.

Alt-text: Unlabelled box

## 1 Introduction

Cervical cancer is largely preventable through treatment of screen-detected cervical lesions. It has a long natural history with a prolonged precancerous phase that is easily detectable and treatable. Exfoliative cytology has been the mainstay for screening of cervical pre-invasive disease (cervical intraepithelial neoplasia or CIN) for almost half a century. HPV testing that detects the presence of high-risk oncogenic HPV (hrHPV) infection is replacing cytology in primary screening programmes. This has been found to have superior performance to that of cytology in the detection of high-grade CIN with better sensitivity and negative predictive value and can provide 60–70% greater protection against cervical cancer in women over the age of 30 [1], [2], [3]. Moreover, hrHPV testing is objective, automated and user-independent ^4^. However, hrHPV testing has a low specificity and triage tests are required to select HPV positive women that should be referred to colposcopy to minimise overburdening of clinical services. Although a number of tests are under investigation, currently cytology is preferred as the test of choice for triage in most settings.

Despite the obvious advantages of HPV testing as a primary screening test, this is costly. Furthermore, triage cytology for hrHPV positive women lacks automation, takes several days to report, is prone to human error and is of low sensitivity [5], [6], [7]. Given the high prevalence of passenger HPV infections with no carcinogenic potential and the mediocre performance of most existing triage tests, novel technologies that offer rapid, affordable and simultaneous HPV testing and automated triaging of women at high risk of high-grade CIN are highly sought after.

Metabolomics involves the detailed investigation of metabolites and small-molecules, which are closely linked to phenotype and can therefore be used to evaluate the health status of a patient ^8^. It has been widely used to study cancer metabolism and identify biomarkers indicative of disease states and underlying aetiology. In the context of cervical cancer and pre-cancer, multiple platforms such as liquid chromatography-mass spectrometry (LC-MS) [9], [10], [11], [12], [13], gas chromatography-mass spectrometry (GC–MS) ^14^, matrix-assisted laser desorption ionisation (MALDI)^15^ and nuclear magnetic resonance (NMR) spectroscopy ^16^ have shown diagnostic potential. However, these methods tend to be laborious, requiring extensive sample preparation or the use of chromatographic techniques prior to mass spectrometry analysis, which prevent their wide use in a clinical setting.

Ambient ionisation mass spectrometry has revolutionised the way metabolomic experiments can be performed as it provides molecular information for a sample in near real-time, without the need for prior sample preparation steps ^17^. Ambient ionisation mass spectrometry has been successfully used for identification of metabolite features that permit diagnosis of different cancers in a variety of tissue types including breast ^18^, liver ^19^, prostate ^20^, kidney ^21^ or brain cancers ^22^. Rapid Evaporative Ionisation Mass Spectrometry (REIMS), included under the “umbrella” term of ambient ionisation techniques, employs commonly-used surgical methods, such as standard electrosurgery or infrared laser surgery, to cause sample evaporation and generate information-rich gas-phase ions ^23^. The tissue-derived smoke that is created from the rapid heating contains information about the phospholipid signature of the tissue which can be used for accurate discrimination of tissue types as well as real-time, intraoperative detection of cancer or evaluation of surgical margins [24], [25], [26]. We have also recently shown that REIMS coupled to electrosurgery via a handheld device (also known as the intelligent knife – iKnife) permits detection of precancerous lesions and cervical cancer with high diagnostic accuracy ^27^.

In order to minimize the user input and facilitate clinical implementation, REIMS has been more recently developed into a high-throughput and automated platform to replace the previous handheld technology. The laser-assisted REIMS (LA-REIMS) setting has been used with success for different applications, such as direct-from-culture identification of bacteria ^28^ or yeast ^29^. A more recent study has also employed LA-REIMS for the diagnosis of Type 2 diabetes in faecal samples ^30^.

In this study, we first evaluated the ability of LA-REIMS to distinguish women with hrHPV infections from those without via analysis of cell pellets recovered from liquid-based cytology (LBC) samples. Further, we aimed to evaluate whether this technology could also detect women with high-grade precancerous cytology.

## 2 Materials and methods

### 2.1 Population – inclusion and exclusion criteria

We prospectively recruited non-pregnant women that attended the colposcopy and gynaecology clinics at Imperial College NHS Healthcare Trust between 2015 and 2018. We recruited a cohort of 65 hrHPV positive women and similar size population of 65 consecutive controls that had tested negative for hrHPV. We included women irrespective of age, parity, ethnicity, smoking habits, the use of contraception and menopause status. We excluded pregnant women and women who were HIV positive. Detailed medical and gynaecological history was collected including time since last sexual intercourse and douching practices. Ethnicity was self-reported as Caucasian, Asian, Black or mixed (Afro-Caribbean/Irish; White African; Afro-Caribbean/British). All women gave written consent (REC Fulham: 13/LO/0126).

### 2.2 Sample collection and preparation

**Fig. 1:**
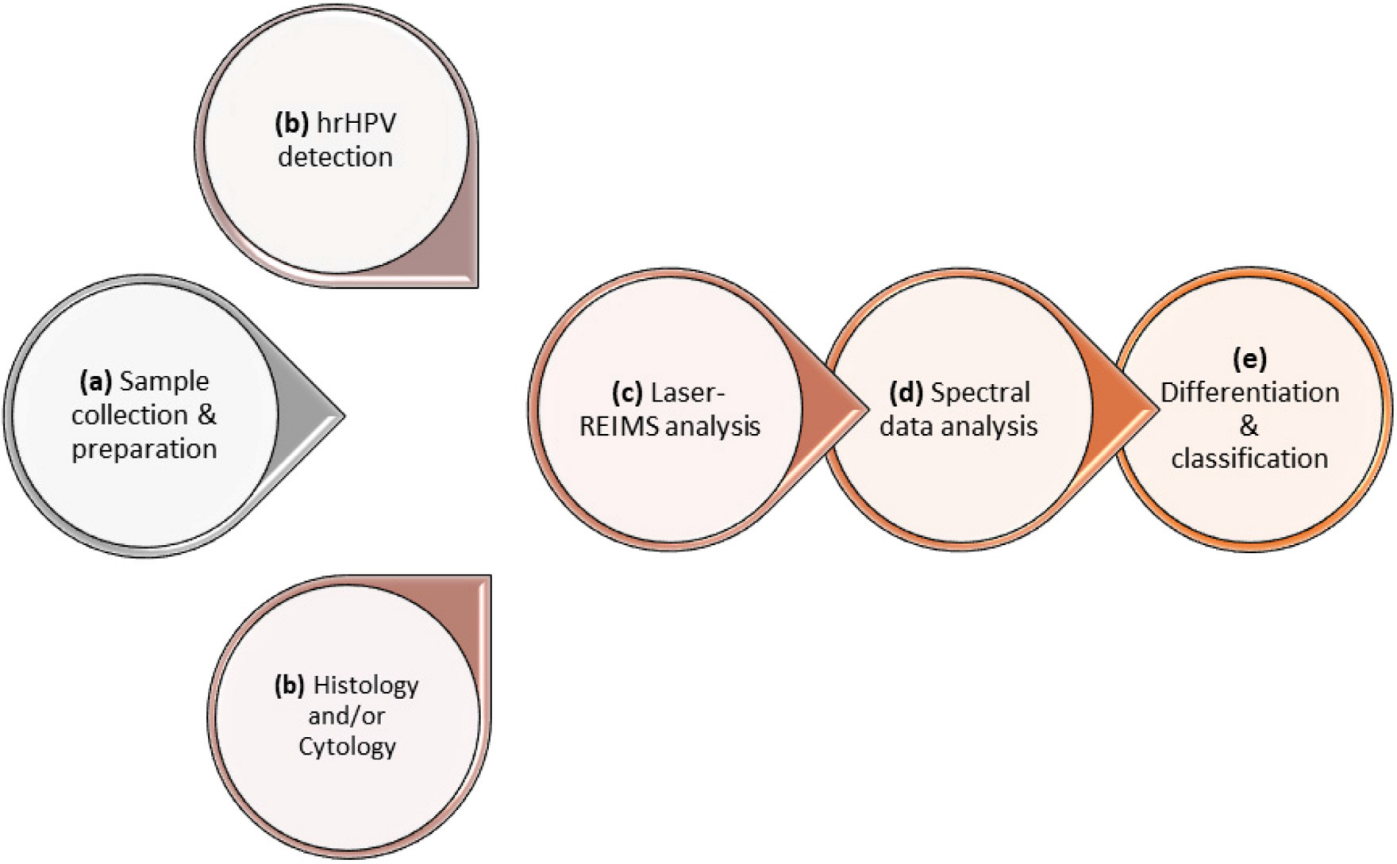
Experimental workflow. **(a)** Liquid-based cytology (LBC) samples are collected and aliquoted for subsequent analyses. **(b)** High-risk (hr) HPV detection along with histological, if clinically indicated, and/or cytological diagnosis are performed. **(c)** LA-REIMS high-throughput analysis is performed, and raw spectral information is collected and pre-processed. **(d)** Analysis of mass spectrometry data is conducted using machine learning approaches. **(e)** The technique's performance for differentiation and classification of the different cohorts is assessed.

From women participating, we collected a liquid-based cytology (LBC) sample of exfoliated cells using a disposable speculum and a cytobrush; the brush was then suspended into the methanol-based preservative solution (ThinPrep, HOLOGIC Inc., USA) and vigorously swirled to release the cytological material and stored at 4 °C until further analysis. The sample was collected without the use of lubrication and prior to the application of acetic acid or iodine. High-risk HPV (hrHPV) detection was performed on the collected samples to identify 14 hrHPV subtypes (16, 18, 31, 33, 35, 39, 45, 51, 52, 56, 58, 59, 66 and 68) using clinically validated hrHPV assays. A brief overview of the method workflow, depicting the steps from sample collection and preparation to spectral generation and classification, is provided in Fig. 1.

From the remaining LBC solution, 2 ml were aliquoted in pre-weighted microcentrifuge tubes and randomised. All samples were prepared and analysed at the same time. The researcher conducting the experiments was blinded to the class of the samples during analysis. Samples were centrifuged at 21,000x*g* for 5 mins and supernatant was removed. Sterile water was used for three sequential washes of the cell pellet, with the supernatant being removed after each centrifuge cycle. The weight of the cell pellet from each sample was recorded prior to analysis with LA-REIMS to ensure the presence of adequate cellular material (>10 mg); cell counting was not feasible in the cell pellet due to the destructive nature of the technique.

### 2.3 High-throughput LA-REIMS analysis

**Fig. 2:**
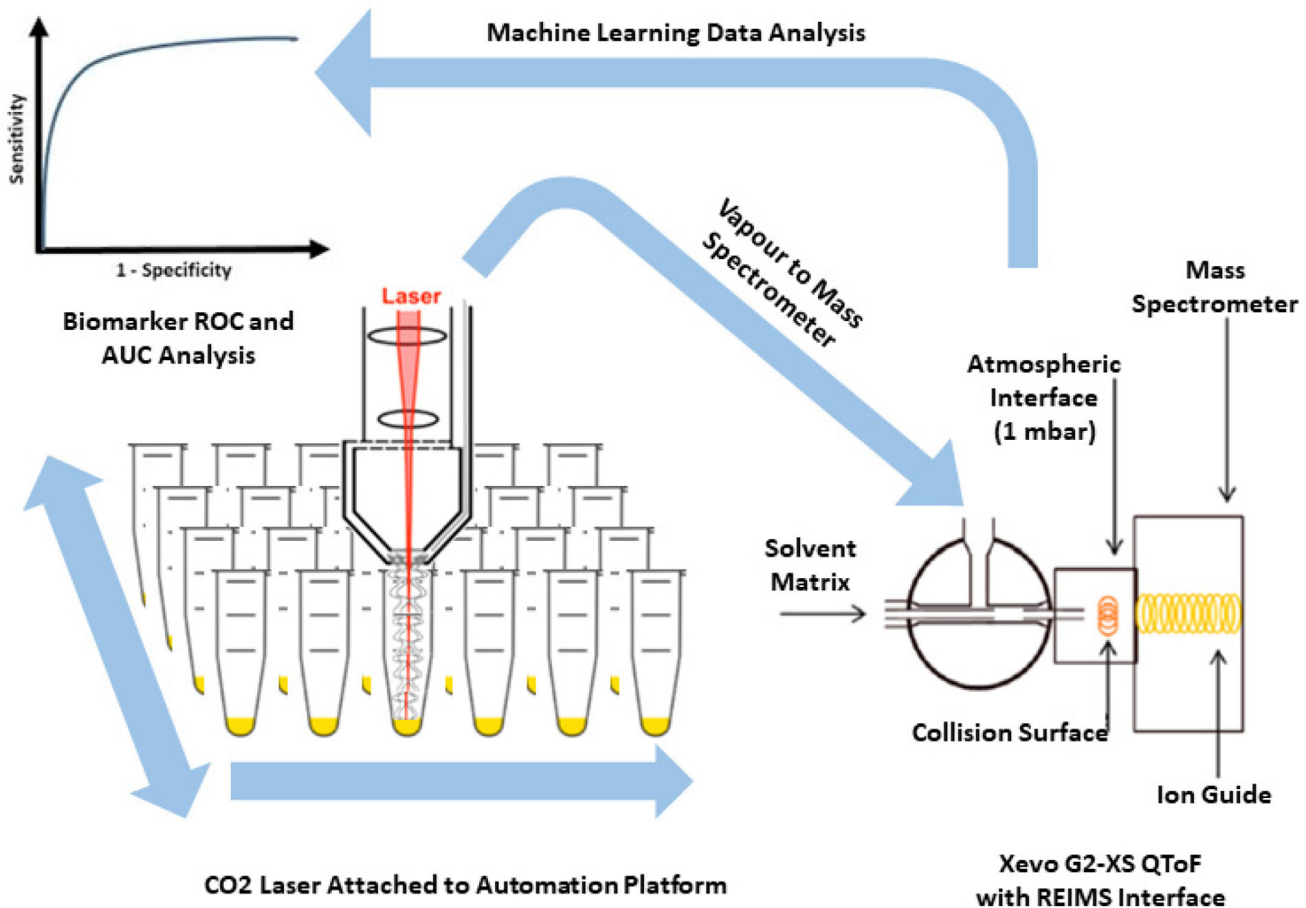
LA-REIMS experimental setting (adapted from Cameron et al. ^31^ and Gowers et al. ^29^).

Pellets of samples were then placed into the high-throughput, commercially available robotic Freedom Evo 75 platform (TECAN, Switzerland) for analysis. A helium gas-cooled CO_2_ surgical laser (FELS-25A, OmniGuide, USA), with a fibre optic beam guide, wavelength of 10.6 μm and 30 psi gas pressure, was utilised with a power setting of 1.5 W in SuperPulse pulsatile mode with 40 ms pulse windows. The resulting analyte-containing vapour was transferred for mass spectrometry analysis through PTFE tubing to a Xevo G2-XS Q-ToF (Waters Corporation, UK) operated under conditions given in Supp Table S1. Prior to entry into the mass spectrometer, the generated vapour was mixed with 2-propanol containing leucine encephalin at a concentration of 0.1 ng/mL, at a flow rate of 0.2 mL per minute. We performed quality control measurements by infusing continuously leucine enkephalin to 2-propanol ionisation matrix that was within an acceptable range; there was no significant difference between the compared groups based on HPV infection and disease severity (Supp Fig. 1). The resulting mixture passed through the REIMS atmospheric interface chamber where solvent−ion clusters collide with a heated (700−800 °C) coil before entry into the StepWave® of the MS instrument ^31^ (Fig. 2). The instrument was calibrated daily using sodium formate in negative ionisation mode, following the manufacturer's standard instructions. Mass spectrometry analysis was performed in full scan, negative ionisation mode with mass spectra acquired over the 50 to 1200 *m/z* range. Representative spectra across the mass range of 50–1000 *m/z* and 600–850 *m/z* are shown in Supp Fig. 2. The required analysis time for running 24 samples was approximately 10 min.

### 2.4 Data and statistical analysis

For the primary analysis, women were classified into two groups: hrHPV positive and negative. We used the resulting spectral data to explore whether LA-REIMS was able to distinguish women with and without a high-risk HPV infection. We calculated accuracy parameters that included sensitivity, specificity and the area under the curve (AUC). Furthermore, we performed additional analysis to explore whether the LA-REIMS had the ability to discriminate women without precancerous changes (normal/HPV) from women with clinically significant disease (CIN2 or worse). We chose this comparison as the most clinically relevant outcome for our main analysis. We performed a series of secondary analysis exploring different comparisons and calculated accuracy parameters for different comparisons. We performed further subgroup analyses using as gold standard histology alone. Finally, we explored the accuracy of LA-REIMS in discriminating normal/HPV from CIN2 or worse as per the main analysis but restricting these to hrHPV positive women in an attempt to mimic the current screening algorithms that uses cytology as a reflex test.

We used histology in preference to cytology to define the disease grade. If histology was present from a punch biopsy and a cone documenting different grade, the most severe lesion was considered. If histology was not available as not clinically indicated (negative or borderline cytology), cytology was used for classification. Cytological and histological assessments were performed by experienced cytopathologists.

To assess whether there were any potential baseline differences in selected clinical and demographic characteristics, we performed statistical analysis using IBM SPSS Statistics (version 26). P values were calculated using a *t*-test or one-way ANOVA test for the age and a Fisher's exact test for hrHPV status, disease status, ethnicity, parity, smoking, contraception and menopause. A *P* value < 0.05 was considered significant.

After mass spectrometric acquisition, all raw spectral data underwent pre-processing with the Offline Model Builder (OMB) software (version 1.1.29.0, Waters) to perform background subtraction and mass drift correction against leucine encephalin lock mass compound (negative ionisation mode *m/z* = 554.2615). Following background subtraction and lockmass correction, spectra were normalised based on total ion count and re-binned to 0.1 Da within the 50 to 1200 *m/z* range. After pre-processing of the mass spectra with the OMB software, filtering for signal-to-noise ratios was completed to identify samples with insufficient cellular material for analysis. This was completed by calculating the average for each of the top ten most intense features in the mass spectra and identifying those samples that were below a single standard deviation in at least three of the features. After sample filtering, a data matrix was exported and uploaded on the MetaboAnalyst 4.0 online platform ^32^, where additional processing was performed for data filtering (mean intensity value), log transformation and Pareto Scaling (mean-centred and divided by the square root of the standard deviation of each variable) prior multivariate analysis. Spectra were further interrogated within the 600–1000 *m/z* range.

Random forest algorithms were used as a machine learning approach for sample classification and evaluation of the technique's performance. Random forest models allow prediction of unknown samples (i.e. test dataset) after training on a known dataset (i.e. train dataset). Receiver operating characteristic (ROC) curves were generated by Monte-Carlo cross validation (MCCV) using balanced sub-sampling. This method is a cross validation approach which creates multiple random splits of the dataset into training and validation data ^33^. For each split, the model is fit to the training data and predictive accuracy is assessed using the validation data. The results are then averaged over the splits. In each MCCV, two thirds (2/3) of the samples were used as the training dataset and the performance of the model was then validated on the one third (1/3) of the samples that were left out, used as the test dataset ^34^.

To tentatively assign the mass spectral features that were most responsible for the observed differentiation, we performed univariate analysis using a volcano plot, which is a combination of fold change and t-tests, (fold change threshold=2; FDR adjusted *P* value with 0.05 threshold). After background removal and mass drift correction in MassLynx software (version 4.1, Waters Corporation), significant mass bins were interrogated to identify peaks within these mass bins with a mass accuracy of two decimal places. The exact mass measures were subsequently used to interrogate the LIPID MAPS database ^35^ and the Human Metabolome Database (HMDB) ^36^, allowing for a mass tolerance of ±0.01 *m/z*, with the highest ranked match being based on the Delta value.

## 3 Results

**Fig. 3:**
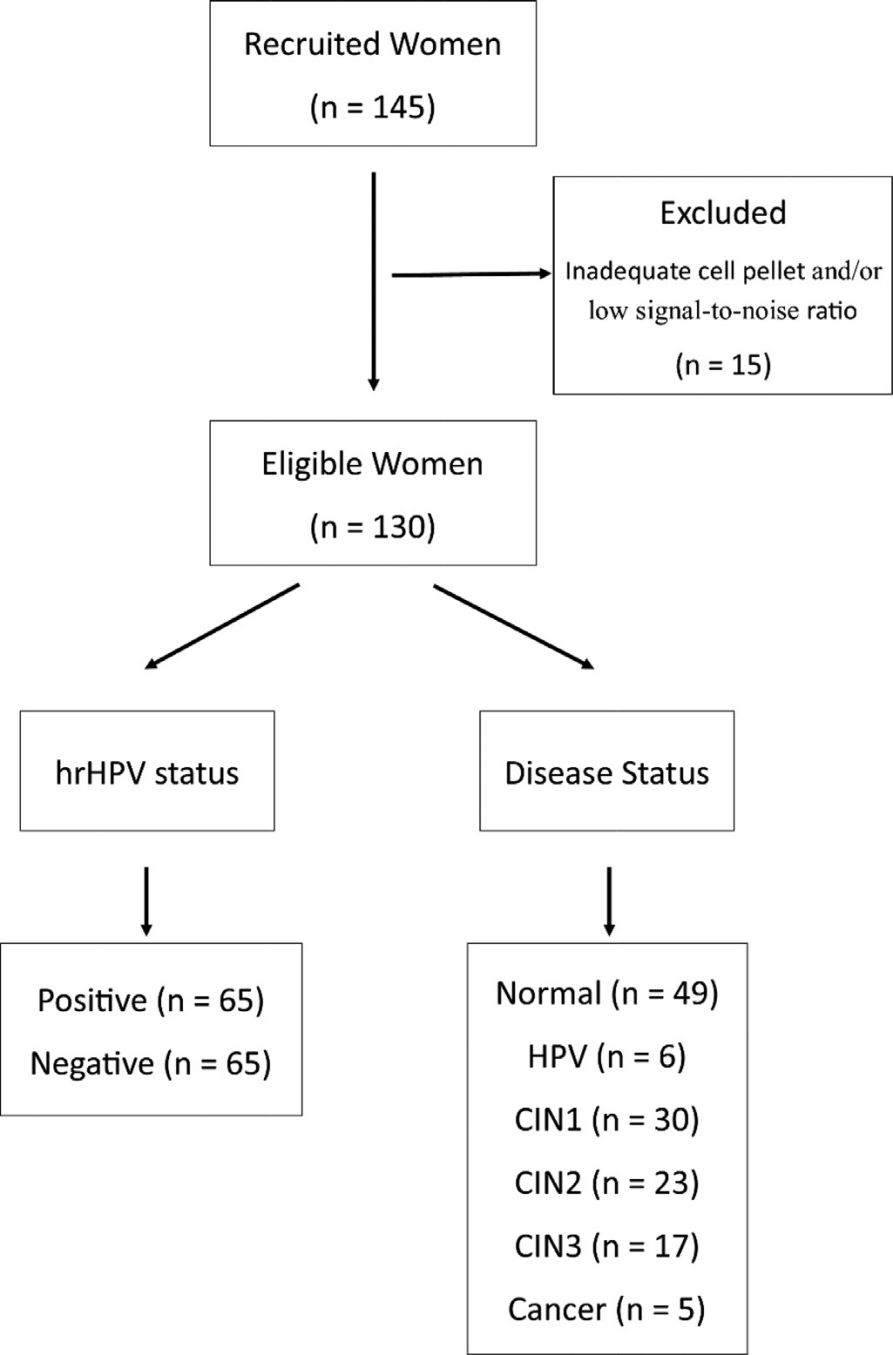
Recruited population - 145 women; 130 eligible for analysis. HPV status: 65 positive – 65 negative. Disease status: Normal = 49 (confirmed by histology (*n* = 32) or cytology (*n* = 17)), HPV = 6, CIN1 = 30, CIN2 = 23, CIN3 = 17 and Cancer = 5. All apart from some normal had histological confirmation of disease status.

We recruited a total of 145 women; 15 were removed from further analysis due to insufficient cellular material (cell pellet weight <10 mg) and/or low signal-to-noise ratio (Fig. 3). Of the remaining 130 women included in the final analysis, half were hrHPV positive (*n* = 65) and half hrHPV negative (*n* = 65) as determined using clinically approved hrHPV assays. The main patient characteristics and demographics of the two patient populations are shown in Table 1. Out of the 65 women positive for hrHPV, 20 (30.8%) had HPV16 only, 3 (4.6%) had HPV18 only, 15 (23.1%) had HPV16 and other hrHPV, 11 (16.9%) had HPV16/18 with or without other hrHPV and 16 (24.6%) had other than 16/18 hrHPV subtypes. The two groups had similar mean age (36.6y for hrHPV positive vs 34.3y for hrHPV negative women) (*P* value=0.139, *t*-test), with the majority being Caucasian in both groups (86 vs 78%) (*P* value=0.077, Fisher's exact test) and nulliparous (71 vs 74%) (*P* value=1, Fisher's exact test). The rate of smokers (28 vs 20%) (*P* value=0.302, Fisher's exact test) and premenopausal women (92 vs 88%) (*P* value=0.719, Fisher's exact test) was higher in the HPV positive group, although the differences were not significant.

hrHPV=high-risk human papillomavirus; CIN=cervical intraepithelial neoplasia

**Table 1:** Patient characteristics of women according to their HPV status (hrHPV+ or hrHPV-). P values were calculated using a *t*-test for the age and a Fisher's exact test for disease status, ethnicity, parity, smoking, contraception and menopause. A *P* value < 0.05 was considered significant.

Patient Characteristics	hrHPV+ (*N* = 65)	hrHPV- (*N* = 65)	*P* value
**Age**			
Mean (SD, range)	36.6 (9.9, 25–69)	34.3 (7.9, 23–64)	0.139
**Disease Status, n/N (%)**			<0.0001
Normal* /HPV	12/65 (18.5)	43/65 (66.2)	
CIN1	13/65 (20.0)	17/65 (26.1)	
CIN2	21/65 (32.3)	2/65 (3.1)	
CIN3	14/65 (21.5)	3/65 (4.6)	
Cancer	5/65 (7.7)	0/65 (0.0)	
**Ethnicity, n/N (%)**			0.077
Caucasian	56/65 (86.2)	51/65 (78.5)	
Asian	6/65 (9.2)	3/65 (4.6)	
Black	3/65 (4.6)	7/65 (10.8)	
Mixed⁎⁎	0/65 (0.0)	4/65 (6.1)	
**Parity, n/N (%)**			1.000
Nulliparous	46/65 (70.8)	48/65 (73.9)	
Parous	16/65 (24.6)	17/65 (26.1)	
Unknown	3/65 (4.6)	0/65 (0.0)	
**Smoking Status, n/N (%)**			0.302
Current smoker	18/65 (27.7)	13/65 (20.0)	
Non/Ex-smoker	44/65 (67.7)	52/65 (80.0)	
Unknown	3/65 (4.6)	0/65 (0.0)	
**Contraception, n/N (%)**			0.653
Nil	24/65 (36.9)	28/65 (43.1)	
Condoms	12/65 (18.5)	11/65 (16.9)	
COCP/POP	16/65 (24.6)	14/65 (21.5)	
IUCD/IUD/Mirena/Contraceptive (injection/depot/implant/patch)	6/65 (9.2)	11/65 (16.9)	
Unknown	7/65 (10.8)	1/65 (1.6)	
**Menopause, n/N (%)**			0.719
Pre-menopausal	60/65 (92.3)	57/65 (87.7)	
Post-menopausal	5/65 (7.7)	3/65 (4.6)	
Unknown	0/65 (0.0)	5/65 (7.7)	

⁎ Normal includes: diagnosis confirmed after histology (*n* = 32) and cytology (*n* = 17).

⁎⁎ Mixed includes: Afro-Caribbean/Irish; White African; Afro-Caribbean/British.

Patient characteristics according to disease status were also examined (Supp Table 2). We included women in the following groups: normal (*n* = 49), HPV (*n* = 6), CIN1 (*n* = 30), CIN2 (*n* = 23), CIN3 (*n* = 17) and cancer (*n* = 5) (Fig. 3). All women had histological confirmation of disease apart from 17 women in the normal controls where cytology defined them as normal controls.

### 3.1 LA-REIMS classification according to high-risk HPV status

**Fig. 4:**
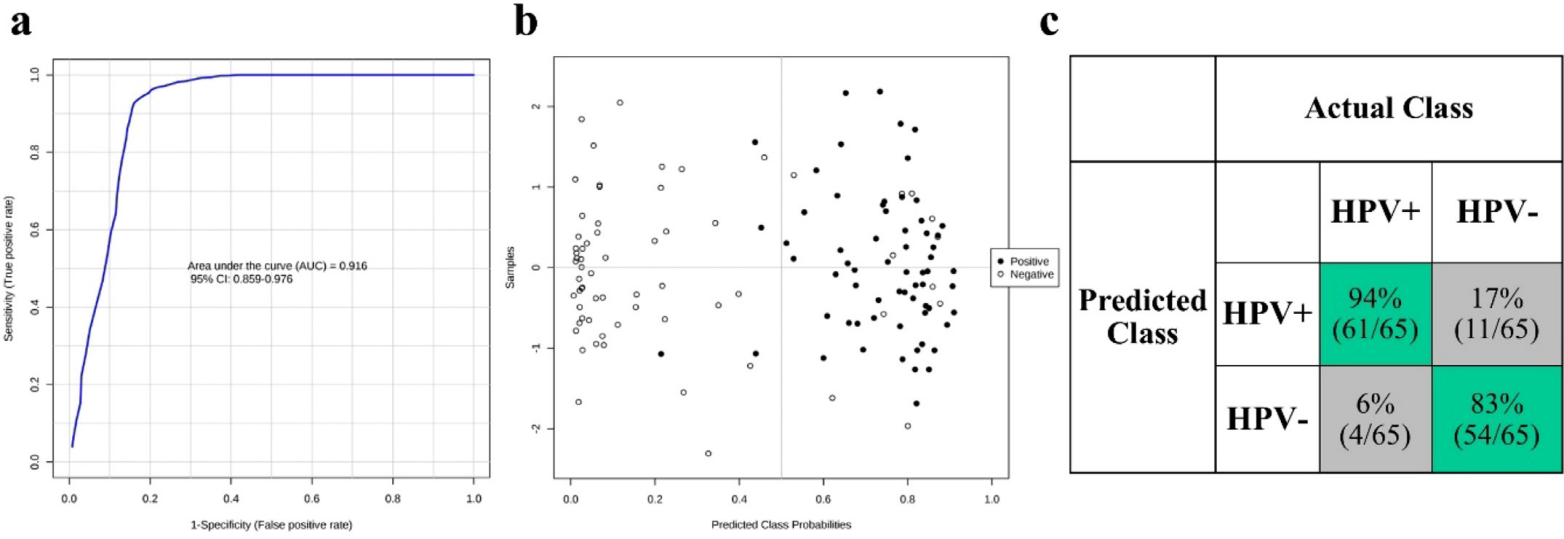
LA-REIMS classification according to hrHPV status. (a) Receiver operating characteristic (ROC) curve after employing random forest as the classification method. The generated values for the area under the curve (AUC) along with 95% confidence intervals (CI) are given within the plot. **(b)** Predicted class probabilities for each sample allowing visualisation of the misclassified samples (hrHPV+ shown as black dots; hrHPV shown as white dots). As a balanced subsampling approach is used for model training, the classification boundary is always at the centre (*x* = 0.5, the dotted line). **(c)** Confusion matrix showing the number of true positives (61/65), true negatives (54/65), false positives (11/65) and false negatives (4/65). Sensitivity and specificity are given in the green-highlighted regions, being 94% and 83% respectively.

The experimental workflow is depicted in Fig. 1. We initially employed multivariate exploratory analysis to assess the diagnostic performance of the automated LA-REIMS platform in distinguishing hrHPV positive from negative women. Fig. 4 shows the classification results after random forest analysis; the receiver operating characteristic (ROC) curve of the best-performing model indicated an area under the curve (AUC) of 0.916 (95% confidence interval CI: 0.859% to 0.976%) (Fig. 4a). The scores plot (Fig. 4b) shows the predicted class probabilities for all samples included in the analysis, indicating correct classification of 61 hrHPV positive and 54 hrHPV negative samples (Fig. 4c). LA-REIMS was able to correctly classify 61 out of 65 hrHPV positive and 54 out of 65 hrHPV negative women, achieving a 94% sensitivity and 83% specificity.

hrHPV: high-risk human papillomavirus

**Fig. 5:**
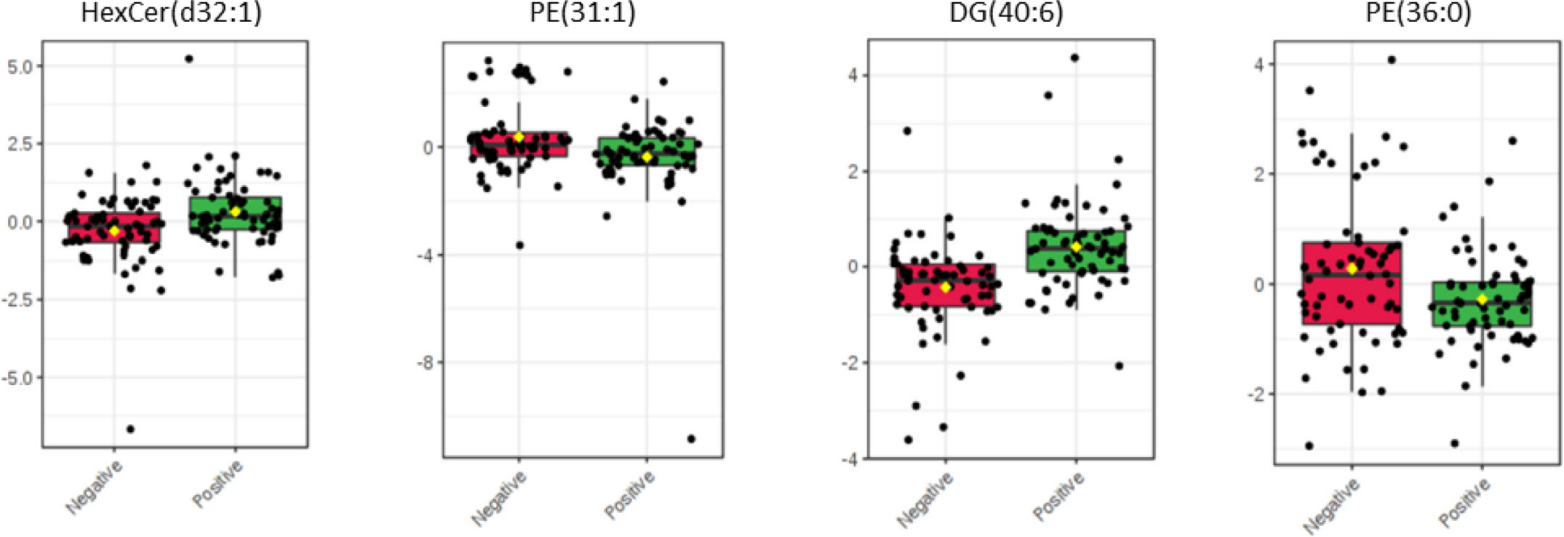
Significant mass spectral features after univariate analysis according to hrHPV status. Important features responsible for the observed segregation between hrHPV negative (*n* = 65) and hrHPV positive (*n* = 65) women were selected by volcano plot with fold change threshold = 2 and FDR adjusted *P* value = 0.05. Tentative assignments of important features were provided after interrogation of the LIPID MAPS database and Human Metabolome Database (HMDB) (Supp Table S3).

Significant and important spectral features that were responsible for the observed differentiation between the two classes were found after univariate analysis and tentatively assigned using online databases (Fig. 5, Supp Table S3). More specifically the following features were found significantly different in our cohort: *m/z* 670.53 (Hexosyl ceramide (HexCer(d32:1)), *m/z* 674.48 (phosphoethanolamine (PE(31:1)), *m/z* 681.55 (diglyceride DG(40:6)) and *m/z* 746.57 (PE(36:0)).

### 3.2 LA-REIMS classification according to cervical disease severity

**Fig. 6:**
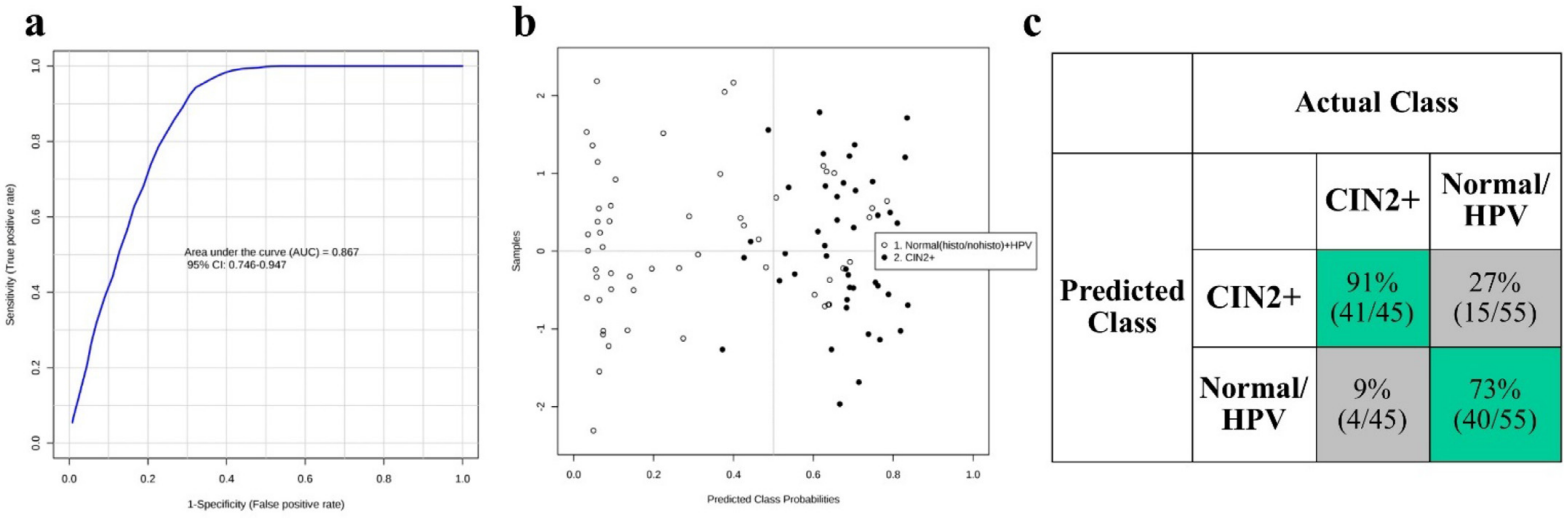
LA-REIMS classification according to cervical disease severity. (a) Receiver operating characteristic (ROC) curve after employing random forest as the classification method. The generated values for the area under the curve (AUC) along with 95% confidence intervals (CI) are given within the plot. **(b)** Predicted class probabilities for each sample allowing visualisation of the misclassified samples (CIN2+ shown as black dots; normal controls shown as white dots). As a balanced subsampling approach is used for model training, the classification boundary is always at the centre (*x* = 0.5, the dotted line). **(c)** Confusion matrix showing the number of true positives (41/45), true negatives (40/55), false positives (15/55) and false negatives (4/45). Sensitivity and specificity are given in the green-highlighted regions, being 91% and 73% respectively.

CIN: cervical intraepithelial neoplasia; HPV: human papillomavirus.

Our secondary analysis aimed to investigate the ability of LA-REIMS to distinguish women with CIN2 or worse (CIN2+) (*n* = 45) from normal controls without preinvasive disease (normal – HPV) (*n* = 55) (Fig. 6), which was considered the most clinically relevant outcome of the main patient groups. After ROC analysis, the achieved AUC was 0.867 (95% CI 0.746–0.947), denoting a satisfactory diagnostic accuracy and trade-off between sensitivity and specificity (Fig. 6a). From the plot of predicted class probabilities (Fig. 6b), it is evident that LA-REIMS was able to correctly classify 41 out of 45 CIN2+ lesions and 40 out of 55 normal controls, allowing for 91% sensitivity and 73% specificity (Fig. 6c).

Additional analyses exploring comparisons of different groups of disease status indicated again that the diagnostic accuracy of LA-REIMS was comparable or even superior to that of reflex cytology (Supp Table S4). We performed further subgroup analyses using as gold standard histology alone in a cohort of 117 women. The results were consistently good: ie. Normal/HPV (*n* = 38) versus CIN2+ (*n* = 45): 85% sensitivity and 66% specificity. Finally, we explored the accuracy of LA-REIMS in discriminating normal/HPV from CIN2 or worse as per the main analysis but restricting these to hrHPV positive women (*n* = 65) in an attempt to mimic the current screening algorithms that uses cytology as a reflex test. This showed good discrimination (normal/HPV (*n* = 12) versus CIN2+ (*n* = 40): 70% sensitivity and 75% specificity.

## 4 Discussion

The introduction of hrHPV in primary screening is expected to lead to new challenges for national screening programmes across the globe. HrHPV test is replacing cytology as a primary screening test in the UK, with reflex triage cytology being used to determine women that need referral to colposcopy ^6^^,^^37^^,^^38^. HrHPV test is anticipated to offer improved sensitivity in detecting high-grade precancer and a 60–70% greater protection against cervical cancer when compared to cytology ^1^, but is less specific and will also increase the number of women who screen positive. hrHPV can be costly, whilst triage cytology is labour-intensive, not automated, subjective and has demonstrated average sensitivity and specificity in detecting CIN2 or worse. Although automated platforms for cytology are currently under investigation (Hologic image-assisted screening (IAS) and Surepath FocalPoint system), these are not widely used in clinical practice*.* To date there has been no single test that has achieved both high sensitivity and specificity in distinguishing women at high risk of high-grade precancer that warrant referral to colposcopy. The identification of innovative technologies that have the potential to offer primary screening and triage of screen-positive women in a single rapid, low cost test that would determine women with transient HPV infection from those that may harbour high-grade pre-invasive remains one of the most significant challenges in the reorganisation of the services in primary cervical screening (Supp Fig 3).

In this study, we have demonstrated that automated LA-REIMS analysis of LBC derived cell pellets can be used to distinguish women with or without a hrHPV infection with high diagnostic accuracy (94% sensitivity and 83% specificity). The emphasis of primary population-based screening is high sensitivity with low false negative rate in order not to miss disease. Our results using the LA-REIMS platform show promise as these preliminary findings document comparable accuracy to other clinically validated molecular HPV tests used for screening [39], [40], [41], [42], but possibly at significantly lower cost (£1.60/sample).

Deregulated lipid metabolism has long been recognised to play an important role in carcinogenesis ^43^. An increased rate of lipid synthesis has been previously described in cancerous samples in comparison to normal controls ^43^^,^^44^, rendering these small molecules promising diagnostic biomarkers. Changes in the lipid content have also been utilised as indicators of tumour progression and metastasis ^45^^,^^46^. The observed lipid alterations reflect structural and functional modifications of the cell membrane which occur during carcinogenesis. Ambient ionisation mass spectrometry techniques favour the detection of small metabolites, such as fatty acids or more complex lipids – a field known as lipidomics ^47^^,^^48^. The use of REIMS, specifically, allows interrogation of the phospholipid signature of a biological sample, which can be subsequently used towards sample identification. REIMS has been previously employed to allow discrimination between healthy and malignant cases based on differences in the relative intensities and distribution of phospholipids [24], [25], [26].

The satisfactory segregation achieved in the current study between women with and without hrHPV infection may be attributed to differential lipid expression due to the viral infection or an altered microbiome associated to HPV. In this study we attempted to tentatively assign discriminatory spectral features, however confirmation of these pilot signatures should be performed in future studies using tandem mass spectrometry. Indeed, infection with HPV has been previously shown to interfere and dysregulate lipid metabolism. In a recent LC-MS study by Ilhan et al. ^49^, HPV-infected individuals were effectively distinguished from HPV-negative women based on perturbations in membrane lipids, which generated characteristic metabolic signatures. A different study, employing a mass spectrometry-based assay for cervical tissue analysis, achieved satisfactory detection of HPV infections in ~80% of the population ^50^. Moreover, a number of previous studies have demonstrated an altered bacterial microbiome in HPV positive women [51], [52], [53], which could also be reflected in our results.

It is known that HPV infection can cause chronic genital inflammation ^54^ and some of the tentatively identified lipids in this study, including sphingolipids and phospholipids, have been previously found to correlate with genital inflammation ^49^. In this previous study by Ilhan et al. ^49^, relative levels of some phospholipids, such as PE, were shown to decrease in an HPV positive cohort when compared to HPV negative women but the differences were not statistically significant. A lipidomic study by Hung et al. ^55^ studying HPV in cervical cancer, also demonstrated differentiation of sphingolipids and phospholipids amongst women with different hrHPV subtypes (altered levels in HPV18 when compared to HPV16 infected women), which shows that HPV infection can alter these metabolites’ levels.

Application of mass spectrometry has been successful in molecular diagnostics of microbial and viral infections [56], [57], [58] as well as metabolic disorders, such as Type 2 diabetes ^30^. Using a REIMS platform, previous studies have detected clinically important bacteria and fungi. More specifically, Cameron et al., reported the ability of REIMS to rapidly identify 153 clinical Candida isolates, allowing 100% correct species classification ^59^. Another study from the same group demonstrated the ability of this setting to characterise clinically important microorganisms from 375 isolates comprising 25 bacterial and fungal species with an overall accuracy of 93.9% ^60^. More recently, a similar laser-assisted REIMS approach using a handheld laser was used for direct metabolomic analysis of human faecal samples allowing the detection of microbiome-level differences ^31^. The LA-REIMS technology has also been used for metabolic phenotyping of individuals with Type 2 diabetes or euglycaemia in faeces, achieving a classification accuracy of 90.5% (89.5% sensitivity and 91.7% specificity) and further highlighting the potential of LA-REIMS for rapid screening and diagnosis of disease ^30^.

As a secondary objective, we assessed the technique's ability to distinguish women according to disease severity based on their spectral information. The sensitivity and specificity achieved for high-grade lesions or worse (CIN2+) were ~90% and ~70% respectively. Reflex cytology has been reported to achieve pooled sensitivity and specificity of 71% and 73% for detection of CIN2+ ^61^, although this varies across different settings and depends on the quality and performance of cytology (sensitivity ranging between 63 and 71% and specificity 73–89% across studies) [61], [62], [63]. Furthermore, Arbyn et al. ^64^ reported that from hrHPV positive women at screening, 56% had ASC-US (atypical squamous cells of undetermined significance) or worse cytology (1604/2882), of whom 612 were found to have CIN3+, corresponding to a positive predictive value (PPV) of 38%. Despite the expected improved accuracy for cytology as a triage test when compared to its performance as screening test given the highest prevalence of the disease ^65^, the sensitivity and specificity is still average. As such, LA-REIMS has the potential to be tested against reflex cytology, the currently preferred triage test in most settings, in future non-inferiority clinical trials.

We chose the classification between normal/HPV versus CIN2+ as the most clinically relevant discrimination for the main analysis and performed a series of alternative comparisons. All confirmed high accuracy in LA-REIMS in discriminating correctly women in each group. When the analysis was restricted to samples with histological confirmation, the results were consistently good. We further mimicked current triage protocols and assessed the ability of LA-REIMS to detect disease state in hrHPV positive women alone with good discrimination, although the sample size was small. A number of hrHPV negative women had precancerous changes in cytology. Classification of cytology in the subgroup of HPV negative women was not performed given the lack of clinical significance of cytology changes in hrHPV negative samples within current screening programmes.

A number of mass spectrometry studies have previously reported satisfactory diagnostic accuracy of cervical cancer or pre-cancer, however these were performed either on cervical tissue samples ^27^, rendering it unsuitable as a screening approach, or using more laborious and time-consuming approaches ^9^^,^[11], [12], [13]. Different studies employing vibrational spectroscopic approaches, including Raman and infrared spectroscopy, have demonstrated the ability of detecting biological differences between normal, precancerous and cancerous cervical pathologies using cytology samples ^66^^,^^67^. Although previous studies have explored the effectiveness LA-REIMS as a new platform of ambient mass spectrometry [29], [30], [31]^,^^60^, this has not been tested in the cervix. To our knowledge, this is the first study of its kind, to determine the use of the robotic, ambient LA-REIMS platform as a means to analyse non-invasively collected cell pellets towards a real-time and objective test for cervical pre-cancer and cancer.

There are of course limitations. The aim of our study was to generate a spectral fingerprint that would allow segregation between the different cohorts (HPV status and cytology). We attempted to tentatively assign discriminatory spectral features. However, biomarker discovery was beyond the scope of this study and, as such, fragmentation spectra were not collected as part of this experimental run. Future studies in larger cohorts should further explore specific biomarkers and biochemical pathways. We used the commonly used methanol-based LBC solution for the suspension of the cells that required sequential washes with water for the methanol removal. Although previous studies showed good biological preservation in alcohol-based fixative solutions^10^^,^^11^^,^^68^^,^^69^, future studies should explore methanol-free preservation media to permit development towards a point-of-care instrument. Even though we chose a representative cohort for this proof-of-concept study, a larger cohort study is required to validate these results and further explore the use of the technology as a triage tool in hrHPV positive women alone. If this confirms high accuracy at least non-inferior to existing tests, follow-on cohort and longitudinal case-control clinical trials nested within existing screening programmes are required prior to implementation in clinical services. Additionally, it should be noted that a biological quality control (QC) was not used in this experiment. Leucine enkephalin levels, infused into the solvent matrix with the analyte vapour, were used to monitor the operation of the REIMS interface and mass spectrometer. However, this is independent from the analysis of biological samples – either through a master mix of randomly selected samples or a commercially available mixture – and means that we did not measure the potential variation of sample heating within and between batches. Although levels of leucine enkephalin are shown to not differ significantly, a potential bias may remain in our analysis that cannot be controlled for as a QC of biological samples was not included.

In conclusion, this is the first study to demonstrate that the automated, high-throughput LA-REIMS platform has the potential to develop into a rapid low-cost platform for screening and triage for cervical cancer. This approach has the advantage of providing simultaneous information for both the hrHPV status (determining if positive or negative for hrHPV) and disease severity (determining if normal or high-grade precancer/cancer), and can permit the accurate selection of screened women that should be referred for further colposcopic assessment with or without biopsy (for diagnosis) and those that can safely return to routine recall with a much-reduced resource requirement with regards to both time and cost. Such a technology could be of particular benefit in middle- or low-income countries given its low cost and the lack of laboratory infrastructure for cytology reporting ^70^. Although LA-REIMS technology, in its present form, could be implemented in central laboratories alone, future adaptation of this technology into a less bulky and user-friendly system, has further potential to permit its use into primary care as a bedside point-of-care tool that would provide instant results minimising patient anxiety. This technology warrants further assessment in well-designed trials.

## Data sharing statement

Raw mass spectral files have been uploaded to the Metabolights repository (MTBLS1371).

## Author contributions

The study was conceived and designed by MK, MP, SJSC and ZT. The samples were collected by SB, MT, AM, AS, AA and MK. MP, SJSC and EW conducted experimental work. Data analysis was performed by MP and SJSC. The data was interpreted, and the manuscript was drafted and revised critically for important intellectual content by all authors. All authors gave final approval of the version to be published and have contributed to the manuscript.

## Declaration of Competing Interest

DAM has a patent EP3570315A3 issued ‘REIMS and DESI analysis of biopsy samples'. ZT reports grants and personal fees from Waters Corporation and has a patent US 9,709,529 issued. Other authors declare no competing interests.

## Appendix Supplementary materials

Image, application 1

## Footnotes

Supplementary material associated with this article can be found, in the online version, at doi:10.1016/j.ebiom.2020.103017.
