# Supplementary material for: Laser-assisted rapid evaporative ionisation mass spectrometry (LA-REIMS) as a metabolomics platform in cervical cancer screening
Source: eBioMedicine. 2020 Sep 25;60:103017. doi: 10.1016/j.ebiom.2020.103017 (PMC7522750; doi:10.1016/j.ebiom.2020.103017)
Supplement: Supplementary file 1 [file mmc1.docx]

# **SUPPLEMENTARY FIGURES**


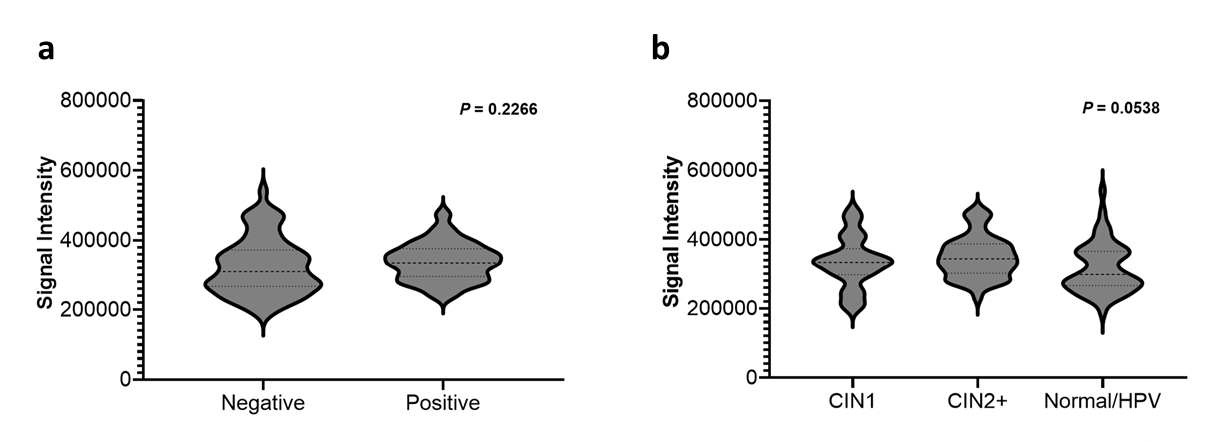


**Supplementary Figure 1:** Quality Control Analysis of Leucine Enkephalin Lock Mass Compound. As a result of sample quantity limitation, the utilization of a biological master mix for quality controls was not possible. However, quality control measurements were made possible as leucine enkephalin, used as an external lock mass compound, was continuously infused to 2-propanl ionisation matrix. The signal intensity of leucine enkephalin was shown to be within an acceptable range and there was no statistically significant difference between the different groups based on: (a) HPV infection status and (b) disease severity.


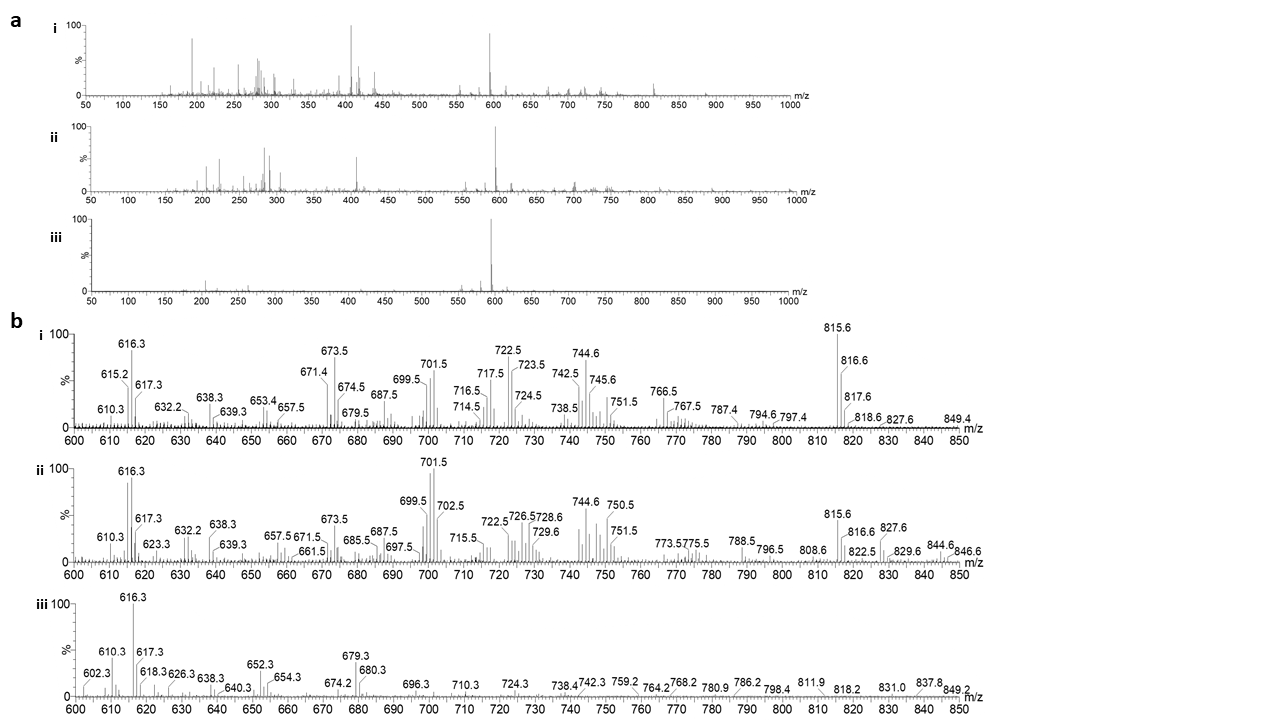


**Supplementary Figure 2:** Representative spectra across the mass range of **(a)** 50-1000 m/z for two samples with adequate cellular material (i-ii) and one sample that was removed due to insufficient cellular material (iii); and **(b)** 600-850 m/z for two samples with adequate cellular material (i-ii) and one sample that was removed due to insufficient cellular material (iii).


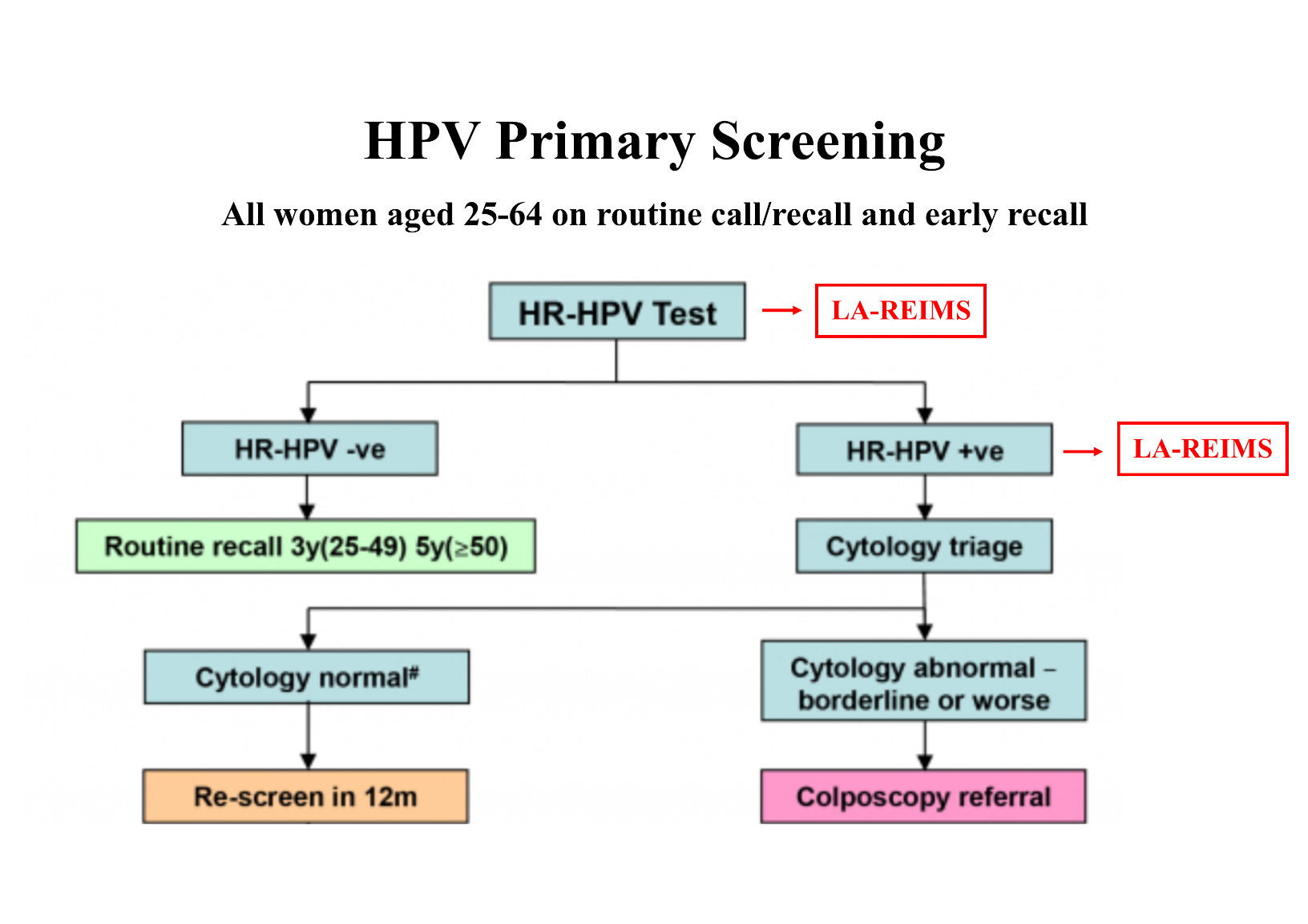


**Supplementary Figure 3:** Current Cervical Screening Algorithms in the UK (<https://phescreening.blog.gov.uk/2017/06/14/what-gps-need-to-know-about-the-introduction-of-primary-hpv-testing-in-cervical-screening/>) and potential applications of LA-REIMS in screening.

# **SUPPLEMENTARY TABLES**

**Supplementary Table S1.** Operational conditions for Xevo G2-XS Q-ToF mass spectrometer.

| **Parameter** | **Setting** |
| --- | --- |
| Scan Time | 1000 ms |
| Scan Mode | Sensitive |
| Mass Analyser | Time of Flight |
| Ionisation Mode | Negative Ion Mode |
| Mass Range | 50 to 1200 |
| Sampling Cone | 80 V |
| Source Offset | 50 V |
| Source Temperature | 100 ^o^C |

**Supplementary Table S2**: Patient characteristics according to disease status. P values were calculated using a one-way ANOVA test for the age and a Fisher’s exact test for hrHPV status, ethnicity, parity, smoking, contraception and menopause. A *P* value < 0.05 was considered significant.

| Characteristics | Normal*/HPV  (N=55) | CIN1  (N=30) | CIN2  (N=23) | CIN3  (N=17) | Cancer  (N=5) | Total  (N=130) | *P* value |
| --- | --- | --- | --- | --- | --- | --- | --- |
| Age |  |  |  |  |  |  | 0.044 |
| Mean  (SD, range) | 35.1  (8.6, 25-64) | 34.9  (8.8, 23-57) | 35.3  (10.4, 26-65) | 34.2  (4.5, 28-44) | 47.6  (12.9, 37-69) | 35.4  (9.0, 23-69) |  |
| hrHPV status, n/N (%) |  |  |  |  |  |  | <0.0001 |
| Positive | 12/55 (21.8) | 13/30 (43.3) | 21/23 (91.3) | 14/17 (82.4) | 5/5 (100) | 65/130 (50.0) |  |
| Negative | 43/55 (78.2) | 17/30 (56.7) | 2/23 (8.7) | 3/17 (17.6) | 0/5 (0.0) | 65/130 (50.0) |  |
| Ethnicity, n/N (%) |  |  |  |  |  |  | 0.155 |
| Caucasian | 41/55 (74.5) | 27/30 (90.0) | 22/23 (95.7) | 14/17 (82.3) | 3/5 (60.0) | 107/130 (82.3) |  |
| Asian | 3/55 (5.5) | 1/30 (3.3) | 1/23 (4.3) | 2/17 (11.8) | 2/5 (40.0) | 9/130 (6.9) |  |
| Black | 7/55 (12.7) | 2/30 (6.7) | 0/23 (0.0) | 1/17 (5.9) | 0/5 (0.0) | 10/130 (7.7) |  |
| Mixed** | 4/55 (7.3) | 0/30 (0.0) | 0/23 (0.0) | 0/17 (0.0) | 0/5 (0.0) | 4/130 (3.1) |  |
| Parity, n/N (%) |  |  |  |  |  |  | 0.005 |
| Nulliparous | 44/55 (80.0) | 21/30 (70.0) | 19/23 (82.6) | 10/17 (58.8) | 0/5 (0.0) | 94/130 (72.3) |  |
| Parous | 11/55 (20.0) | 8/30 (26.7) | 4/23 (17.4) | 5/17 (29.4) | 5/5 (100) | 33/130 (25.4) |  |
| Unknown | 0/55 (0.0) | 1/30 (3.3) | 0/23 (0.0) | 2/17 (11.8) | 0/5 (0.0) | 3/130 (2.3) |  |
| Smoking Status, n/N (%) |  |  |  |  |  |  | 0.245 |
| Current smoker | 9/55 (16.4) | 8/30 (26.7) | 6/23 (26.1) | 6/17 (35.3) | 2/5 (40.0) | 31/130 (23.8) |  |
| Non/Ex-smoker | 46/55 (83.6) | 21/30 (70.0) | 17/23 (73.9) | 9/17 (52.9) | 3/5 (60.0) | 96/130 (73.9) |  |
| Unknown | 0/55 (0.0) | 1/30 (3.3) | 0/23 (0.0) | 2/17 (11.8) | 0/5 (0.0) | 3/130 (2.3) |  |
| Contraception, n/N (%) |  |  |  |  |  |  | 0.447 |
| Nil | 29/55 (52.7) | 8/30 (26.7) | 6/23 (26.1) | 6/17 (35.3) | 3/5 (60.0) | 52/130 (40.0) |  |
| Condoms | 11/55 (20.0) | 5/30 (16.7) | 4/23 (17.4) | 2/17 (11.8) | 1/5 (20.0) | 23/130 (17.7) |  |
| COCP/POP | 9/55 (16.4) | 9/30 (30.0) | 8/23 (34.8) | 4/17 (23.5) | 0/5 (0.0) | 30/130 (23.1) |  |
| IUCD/IUD/Mirena/  Contraceptive (injection/depot/implant/patch) | 6/55 (10.9) | 6/30 (20.0) | 2/23 (8.7) | 2/17 (11.8) | 1/5 (20.0) | 17/130 (13.1) |  |
| Unknown | 0/55 (0.0) | 2/30 (6.6) | 3/23 (13.0) | 3/17 (17.6) | 0/5 (0.0) | 8/130 (6.1) |  |
| Menopause, n/N (%) |  |  |  |  |  |  | 0.528 |
| Pre-menopausal | 47/55 (85.4) | 28/30 (93.3) | 21/23 (91.4) | 17/17 (100) | 4/5 (80.0) | 117/130 (90.0) |  |
| Post-menopausal | 4/55 (7.3) | 2/30 (6.7) | 1/23 (4.3) | 0/17 (0.0) | 1/5 (20.0) | 8/130 (6.2) |  |
| Unknown | 4/55 (7.3) | 0/30 (0.0) | 1/23 (4.3) | 0/17 (0.0) | 0/5 (0.0) | 5/130 (3.8) |  |

* Normal includes: diagnosis confirmed after histology (n=32) and cytology (n=17)

** Mixed includes: Afro-Caribbean/Irish; White African; Afro-Caribbean/British

**Supplementary Table S3:** Tentative assignments of statistically significant spectral features after univariate analysis. The exact mass measures were used to interrogate the LIPID MAPS database and the Human Metabolome Database (HMDB), allowing for a mass tolerance of ±0.01 *m/z*, with the highest ranked match being based on the Delta value.

| **Exact Mass (m/z)** | **Matched Mass (m/z)** | **Database** | **Delta** | **ppm** | **Name** | **Formula** | **Ion** |
| --- | --- | --- | --- | --- | --- | --- | --- |
| 670.53 | 670.5264 | LIPID MAPS | 0.0036 | 5 | HexCer(d32:1) | C_38_H_73_NO_8_ | [M-H]- |
| 674.48 | 674.4766 | LIPID MAPS & HMDB | 0.0034 | 5 | PE(31:1) | C_36_H_70_NO_8_P | [M-H]- |
| 681.55 | 681.5463 | HMDB | 0.0037 | 5 | DG(40:6) | C_44_H_74_O_5_ | [M-H]- |
| 746.57 | 746.5705 | LIPID MAPS & HMDB | 0.0005 | 1 | PE(36:0) | C_41_H_82_NO_8_P | [M-H]- |

**Supplementary Table S4.** Secondary and Subgroups Analyses according to disease status. **(a)** Secondary analysis exploring the discrimination for different comparisons of disease state showing good discrimination. **(b)** Subgroup analysis including only samples with histological confirmation showing consistently high discrimination. **(c)** Subgroup analysis of hrHPV positive women alone showing discrimination ability similar or superior to that of reflex cytology (histological confirmation for all women).

| **Comparison Groups** | | **Sensitivity (%)** | **Specificity (%)** |
| --- | --- | --- | --- |
| **a. All* (N=130)** | |  |  |
| Normal/HPV (n=55) | CIN/Ca (n=75) | 87 | 63 |
| Normal/HPV (n=55) | CIN (n=70) | 80 | 65 |
| Normal/HPV/CIN1 (n=85) | CIN2 or worse (n=45) | 87 | 65 |
| Normal (n=49) | HPV or worse (n=81) | 80 | 66 |
| Normal (n=49) | CIN2 or worse (n=45) | 93 | 73 |
| Normal (n=49) | CIN2/CIN3 (n=40) | 95 | 73 |
| **b. Histology only (N=117)** | |  |  |
| Normal/HPV (n=38) | CIN2/CIN3 (n=40) | 80 | 65 |
| Normal/HPV (n=38) | CIN2 or worse (n=45) | 85 | 66 |
| Normal/HPV/CIN1 (n=68) | CIN2 or worse (n=45) | 89 | 60 |
| **c. HrHPV+ (N=65)** | |  |  |
| Normal/HPV (n=12) | CIN2 or worse (n=40) | 70 | 75 |

*Histological diagnosis in all but 17 normal samples considered normal on the basis of a normal cytology
